# Dynamic Complexes in the Chaperonin-Mediated Protein Folding Cycle

**DOI:** 10.3389/fmolb.2016.00080

**Published:** 2016-12-08

**Authors:** Celeste Weiss, Fady Jebara, Shahar Nisemblat, Abdussalam Azem

**Affiliations:** George S. Weiss Faculty of Life Sciences, Department of Biochemistry and Molecular Biology, Tel Aviv UniversityTel Aviv, Israel

**Keywords:** chaperonin, GroEL, GroES, protein folding, football, symmetric, chaperone

## Abstract

The GroEL–GroES chaperonin system is probably one of the most studied chaperone systems at the level of the molecular mechanism. Since the first reports of a bacterial gene involved in phage morphogenesis in 1972, these proteins have stimulated intensive research for over 40 years. During this time, detailed structural and functional studies have yielded constantly evolving concepts of the chaperonin mechanism of action. Despite of almost three decades of research on this oligomeric protein, certain aspects of its function remain controversial. In this review, we highlight one central aspect of its function, namely, the active intermediates of its reaction cycle, and present how research to this day continues to change our understanding of chaperonin-mediated protein folding.

## Introduction

Extensive studies carried over the years to uncover the mechanism behind functioning of the bacterial GroEL/GroES chaperonins led to a generally accepted description of their pathway of operation. The individual components that assemble to form the active complexes have been crystallized and, the interactions that mediate formation of the complexes have been clearly described. Yet, due to the highly dynamic nature of the system, many aspects of their operation remain obscure, and conflicting models describing their function are endorsed. Major controversy in the field is related to nature of the active species in the chaperonin-mediated protein folding cycle: Is it really a case of mutually exclusive models, as many think i.e., is the active form either a symmetrical complex (American football-like complex) or an asymmetric complex (bullet-shaped complex)? Are there additional factors that affect the active species? Are there additional species that participate in the cycle? The discovery of divergent chaperonins in chloroplast and mitochondria has added an additional dimension to this discussion. Do all type I chaperonins operate utilizing the same functional mechanism? In this review, we present the evolution of our understanding of the chaperonin cycle and attempt to convey the fine differences between the two major views of the GroEL–GroES reaction mechanism. We also show how the study of organellar chaperonins can contribute to our understanding of the mechanism by which type I chaperonins carry out their protein folding function.

## The key players

**Figure 1:**
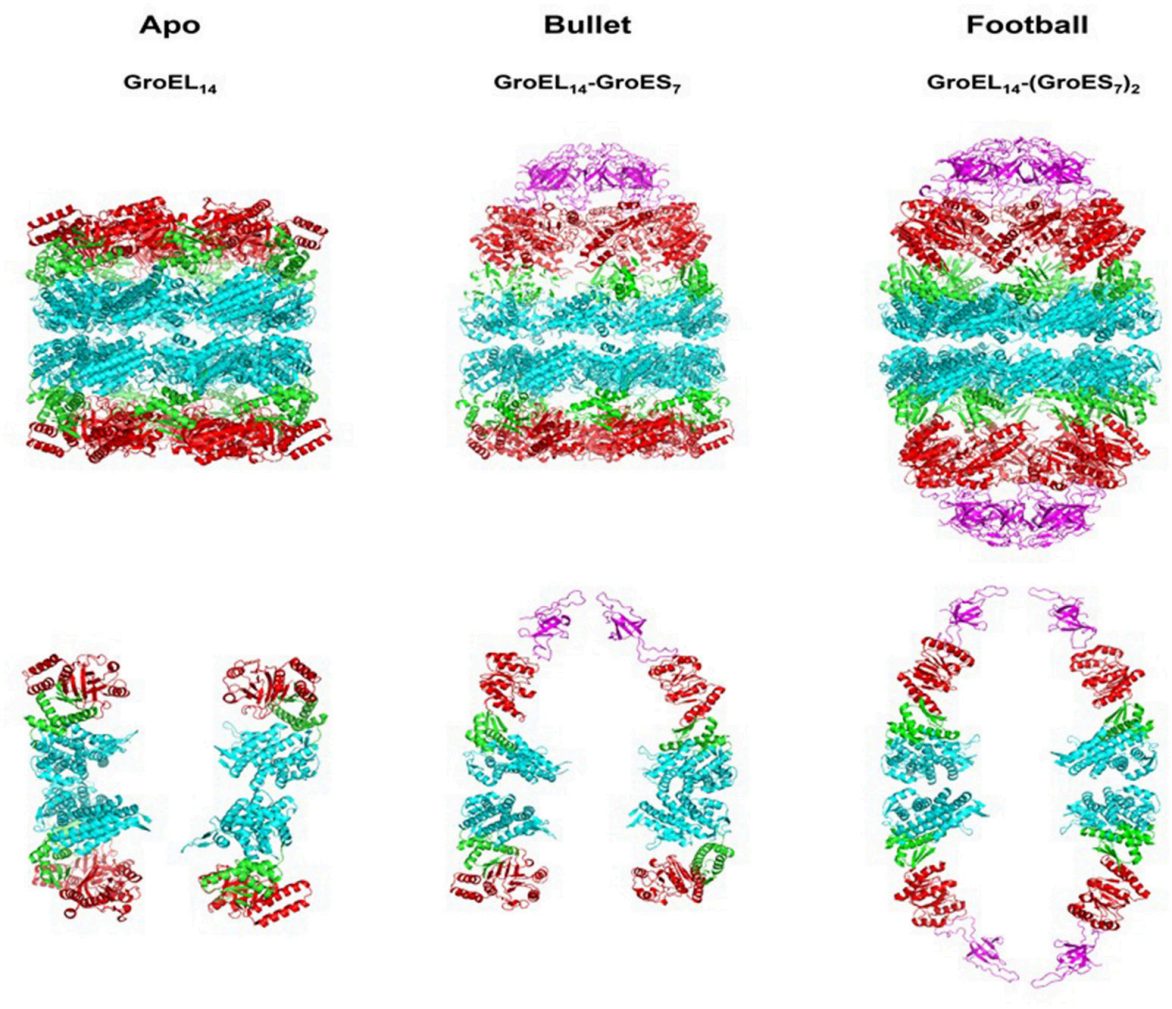
**Crystallographic models showing the architecture of the major chaperonin complexes**. Left figure, unliganded, apo GroEL_14_, PDB code 4WGL; Center figure, GroEL_14_ with one bound GroES_7_ co-chaperonin (“bullet”), PDB code 1AON; right figure, GroEL_14_ with two bound GroES co-chaperonin heptamers (“football”), PDB code 4PKO. The GroES co-chaperonin is colored purple. The three domains of each GroEL subunit are color coded as follows: Apical domain, red; Equatorial domain, cyan; Intermediate domain, green. The top row of figures shows the full structure of each oligomer. The bottom row presents two subunits of each ring, in order to better visualize the spatial orientation of each subunit and its domains. The figure was generated using the PyMOL program (The PyMOL Molecular Graphics System, version 1.5.0.4; Schrödinger, LLC; available at www.pymol.org).

The name chaperonins was coined almost three decades ago to describe the 60 kDa heat shock protein family, a group of ubiquitous proteins that share primary sequence homology, in some cases as low as 20–30% (Hemmingsen et al., 1988; Hill and Hemmingsen, 2001). They are divided into two groups: type I chaperonins and type II chaperonins. The latter is found in the eukaryotic cytosol (CCT and TCP-1) and Archaea, while type I is located in bacteria, mitochondria, and chloroplasts (Hill and Hemmingsen, 2001). The primary role of chaperonins is to prevent aggregation of nascent and misfolded polypeptides and ultimately facilitate their correct (re) folding (Goloubinoff et al., 1989a,b; Horwich et al., 2007; Saibil et al., 2013; Hayer-Hartl et al., 2016). How this occurs is still not completely understood and is the topic of much debate (Jewett and Shea, 2010), however, accumulating evidence suggests that in the case of misfolded proteins, the chaperonin exerts an unfoldase action on the protein, overcoming the free energy barrier (Todd et al., 1996; Walter et al., 1996; Finka et al., 2016). In addition, to the major protein-folding activities, moonlighting functions were also reported for plant and various bacterial systems harboring multiple chaperonin homologs (Lund, 2009; Henderson et al., 2013; Vitlin Gruber et al., 2013; Fares, 2014). The most widely studied prototype at the mechanistic level is the GroEL chaperonin of *Escherichia coli*. Its ~60 kDa subunits assemble into barrel-shaped structures built of two heptameric rings (Hendrix, 1979; Höhn and Wuttke, 1979; Braig et al., 1994; Xu et al., 1997) composed of identical subunits. Each subunit contains three functional domains: the equatorial domain, site of the ATP binding pocket; the apical domain, which binds substrate and GroES; the intermediate domain, which connects the previous two and allows for dynamic structural changes within the molecule (Figure 1). The tetradecameric cylinders harbor the binding sites for unfolded/misfolded substrate proteins, which reside inside the barrel lumen (the Anfinsen cage; Buckle et al., 1997; Chaudhuri and Gupta, 2005; Chen et al., 2013). Due to its double ring assembly, each GroEL molecule can bind two substrate molecules with high affinity (Viitanen et al., 1992; Llorca et al., 1997; Taguchi et al., 2004). In the absence of necessary co-factors, some substrate proteins can bind tightly to the GroEL molecule for extended periods of time in an unfolded conformation (Goloubinoff et al., 1989a; Viitanen et al., 1992; Hartman et al., 1993; Hartmann and Eisenstein, 2000). The folding reaction proceeds through multiple steps, during which the chaperone undergoes major ordered and concerted conformational changes (Hartman et al., 1993; Weissman et al., 1994). The driving force for these conformational changes, as well as their timing, is provided by ATP hydrolysis and the binding of the co-chaperonin GroES (Todd et al., 1994). The latter is itself an oligomeric protein, which assembles into a single heptameric ring arranged in a dome-like structure (Hunt et al., 1996; Mande et al., 1996).

## The major complexes

**Figure 2:**
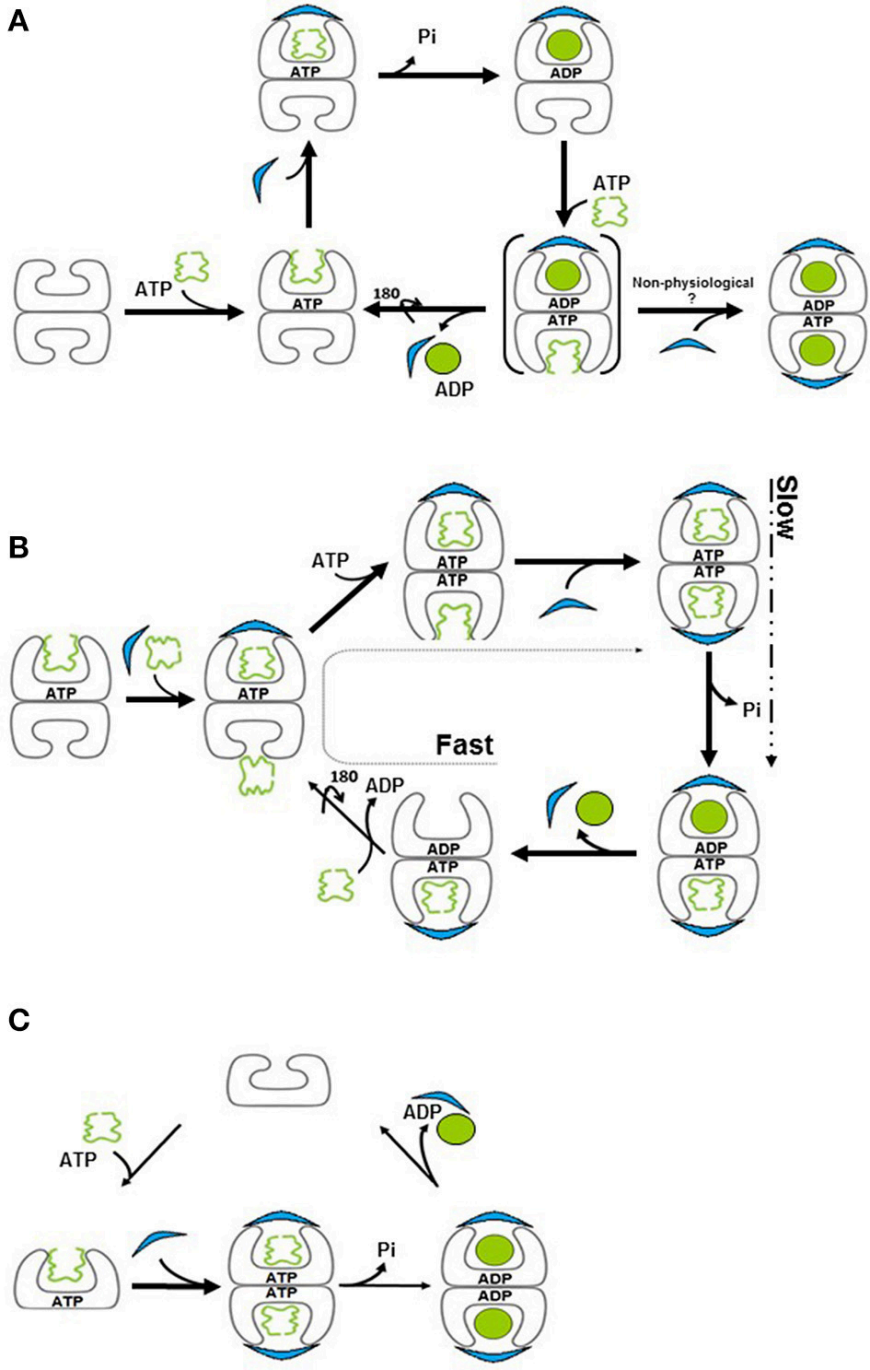
**Models for the chaperonin reaction cycle. (A)** Unfolded protein binds to the apo (“brick”) form of GroEL and is capped by GroES in the presence of ATP, forming the “*cis*” ring. Binding of ATP to the opposite, “*trans*” ring induces release of GroES, ADP and folded protein from the “cis” ring, such that protein folding cycles between one side and the other. Brackets signify a transient species. **(B)** In the presence of substrate protein, ADP to ATP exchange is extremely rapid, resulting in formation of, a symmetric “football” intermediate, in which protein folding takes place simultaneously in both rings. ATP hydrolysis is now the slower, rate-limiting step, resulting in the accumulation of the football form. This form reverts briefly to a bullet conformation upon ATP hydrolysis. **(C)** The mitochondrial chaperonin exists in equilibrium between single- and double-ringed forms. Upon binding of ATP and GroES, the equilibrium is shifted to the double-ringed form. Protein folding takes place in both chambers and release of the cochaperonin transpires upon ATP hydrolysis.

Early after the discovery of chaperonins, it became clear that modulation of GroEL activity is governed by complex formation with GroES, which occurs only following nucleotide-induced conformational changes in the GroEL oligomer (Goloubinoff et al., 1989a,b; Roseman et al., 2001). This discovery was followed by extensive research aimed at identifying the active form of the GroEL–GroES complex. In their pioneering study, Langer and coworkers used EM to identify two forms of the chaperonin *in vitro*: the apo form, consisting of the GroEL tetradecamer alone, without GroES, and a complex containing one tetradecamer of GroEL bound to one GroES heptamer, formed in the presence of ADP (Langer et al., 1992). This form was suggested to be the active form of the system and became known as the asymmetric, bullet-shaped complex (Langer et al., 1992). Subsequently, a third chaperonin complex was observed in the presence of ATP, by several groups (Azem et al., 1994b; Harris et al., 1994; Llorca et al., 1994; Schmidt et al., 1994). The third form is composed of one GroEL barrel sandwiched in between two GroES heptamers, in a symmetric complex, known as the “football” (American)—like complex. High-resolution crystal structures were obtained for all three forms over the years (Figure 1) (Braig et al., 1994, 1995; Boisvert et al., 1996; Xu et al., 1997; Chen and Sigler, 1999; Bartolucci et al., 2005; Fei et al., 2013, 2014; Koike-Takeshita et al., 2014). In these studies, contacts between the subunits within rings and between GroEL/GroES oligomers have been delineated. More importantly, structural changes that occur during the reaction cycle have also been elucidated, through the analysis of various nucleotide-bound forms (Roseman et al., 1996, 2001; Ranson et al., 2001, 2006; Clare et al., 2009, 2012). It has become clear from the vast number of studies that the system is very dynamic in the presence of ATP, and what we are able to capture at any one point, in the test tube, may not necessarily reflect the only active form of the reaction (Todd et al., 1994; Yang et al., 2013; Taguchi, 2015; Yamamoto and Ando, 2016). Indeed, the concentration and type of nucleotide, the presence of mono- and divalent cations and other parameters may determine the form of the complex that is detected and efficiency of protein folding activity (Todd et al., 1993; Azem et al., 1994a, 1995; Diamant et al., 1995; Engel et al., 1995). In a single cycle of ATP hydrolysis, GroEL will bind one or two substrate protein monomers, bind one or two GroES heptamers, bind and hydrolyze 14 ATP, fold the substrate protein, and eject the bound components, all in a matter of seconds (Figure 2). What we observe in the standard biophysical examination is the steady state levels of the complexes with a strong bias for the rate-limiting complex of the cycle under the tested conditions (Todd et al., 1994; Fei et al., 2013; Yang et al., 2013; Taguchi, 2015; Yamamoto and Ando, 2016).

## The reaction cycle

If the forms that we observe in the test tube do not necessarily reflect the only present or active ones, how can we accurately map out the reaction cycle of the system? The answer to this question comes from numerous kinetic and mechanistic studies (for a review see Skjærven et al., 2015; Taguchi, 2015) that enable us to peek into what is really happening in order to identify shorter-lived complexes. To simplify the arguments, we will focus on events that occur in the presence of unfolded substrate protein. Assuming that we have initiated the cycle with the simplest component, the apo GroEL, then the next step will be binding of ATP and/or substrate protein followed by GroES binding, which leads to formation of the folding-competent form. What follows this step constitutes the crux of the controversy. The canonical view suggested that the complex moves through the asymmetric “bullet” cycle (Figure 2A) (Horwich et al., 2006; Hayer-Hartl et al., 2016) while an alternative understanding suggested that the reaction proceeds via the symmetric “football” cycle (Figure 2B) (for reviews see Grallert and Buchner, 2001; Taguchi, 2015).

In the first model, the GroEL tetradecamer alternates between the bullet complex and the apo form, complexed with nucleotide. An important feature of this mechanism is the sequential nature, by which binding of ATP and substrate protein to the *trans* ring stimulates release of GroES, ADP and sequestered substrate from the *cis* ring (Rye et al., 1999). According to this model, the strong negative cooperativity in nucleotide binding between the two GroEL rings (Gruber and Horovitz, 2016) ensures that nucleotide binding to one ring will suppress nucleotide binding and hydrolysis in the opposing ring (Horwich et al., 2007). Thus, a complex with nucleotide and GroES bound on both sides will not form. For many years, this model was almost universally accepted as that which accurately describes the GroEL reaction cycle.

In an alternative model, known as the symmetrical “football” model, the complex alternates between the symmetric complex and the asymmetric form. Despite the negative cooperativity in nucleotide binding that exists between the two rings, conformations with ATP occupying both rings have been described (Clare et al., 2012), along with numerous reports of football structures, which have GroES bound to both sides (Azem et al., 1994b; Harris et al., 1994; Llorca et al., 1994; Schmidt et al., 1994). The involvement of these species in refolding was inferred from many early kinetic studies on GroE-mediated refolding to their native state of foldable substrates such as Rubisco, mMDH, and a maltose binding protein variant, all of which demonstrated a clear correlation between the efficiency of refolding and the occurrence of symmetric GroEL14/GroES14 complexes (Azem et al., 1995; Sparrer et al., 1997; Ben-Zvi et al., 1998; Beissinger et al., 1999).

Were the symmetric complexes to represent a side-abortive reaction or dead end, then one would not expect to see such a correlation, rather, the opposite of what was observed. This correlation was substantiated by sophisticated mechanistic studies demonstrating the importance of the symmetric intermediate in the protein folding cycle (Koike-Takeshita et al., 2008; Sameshima et al., 2010a; Takei et al., 2012; Yang et al., 2013; Ye and Lorimer, 2013; Fei et al., 2014; Yamamoto and Ando, 2016).

## Recent developments and outstanding questions

Earlier studies showed that in the presence of substrate, the chaperonin complex behaves differently than in its absence (Motojima and Yoshida, 2003; Motojima et al., 2004). Further investigation demonstrated that substrate protein facilitates the formation of symmetric, football complexes (Sameshima et al., 2010a). Recent studies using FRET-based analyses concluded that the substrate protein accelerates ADP exchange, in the complex (Ye and Lorimer, 2013; Fei et al., 2014). Thus, the football model posits that if we follow the kinetics of formation and dissociation of cycle intermediates, we will find that both exist in solution (symmetrical and asymmetrical complexes). However, when we use steady state analyses to detect complexes, the form that precedes the rate-limiting step is that which will primarily be observed. Since ADP exchange in the presence of substrate protein occurs very fast relative to ATP hydrolysis, the major species observed in the presence of substrate protein is the football (Takei et al., 2012; Ye and Lorimer, 2013; Iizuka and Funatsu, 2016; Figure 2B). In the absence of substrate protein, the rate-limiting step is the release of ADP, leading to population of the species preceding this step, the asymmetric form.

Is function of the two rings coordinated or do they function as independent folding chambers? Consistent with conclusions of early kinetic studies, single-molecule analyses demonstrate that the first GroES to interact with GroEL is not necessarily the first one to dissociate from the symmetric complex. Rather, the dissociation may occur randomly (Corrales and Fersht, 1996; Sameshima et al., 2010b). A new study using state of the art AFM to dissect molecular events related to GroES binding revealed that that inherently different types of football species can exist, and they will alternate or not, in release of GroES, depending upon the nature of the specific football species (Yamamoto and Ando, 2016). The authors postulate that complete exchange of seven ADPs with seven ATPs ensures that the system goes through an alternating pathway, while incomplete exchange of nucleotide at the *trans*-ring may cause the cycle to go through a non-alternating pathway in which the newly bound GroES dissociates first.

Although the above studies suggest that GroEL may function as two independent folding chambers, a number of facts indicate that the picture is not entirely clear. Firstly, why would such an elaborate system of cooperativity be conserved in *E. coli* if it is not essential? In the classic model, negative cooperativity is taken to its extreme, so that nucleotide binding on one ring completely precludes binding in the opposing ring (Horwich, 2011). But perhaps the effect is not so drastic. In fact, when initial rates of ATP hydrolysis were measured in GroEL as a function of ATP concentration, two transitions were observed, with respective midpoints of 16 and 160 μM (Yifrach and Horovitz, 1995). This data suggests that, despite negative cooperativity, both sides are expected to be saturated with nucleotide under most experimental or cellular conditions. Even in the presence of 0.5 mM ADP (which is inhibitory for refolding and prevents football formation) and 1.5 mM ATP, a majority of football species was observed, which would require that nucleotide be bound to both rings (Azem et al., 1995). However, it is still possible that negative cooperativity retained in this structure, may contribute to alternating release of GroES, resulting in a more efficient machine. This would be consistent with the fact that the majority of GroES release was shown to occur via polarity change (69%) by way of a football complex (Yamamoto and Ando, 2016). Another reason for retaining such a cooperative system could be the fact that GroEL is able to fold large proteins that cannot be accommodated inside the cavity underneath the GroES (Chaudhuri et al., 2009; Dahiya and Chaudhuri, 2014; Pastor et al., 2016). In this instance, it is possible that release of the *cis*-bound substrate must be induced by *trans* binding of substrate, ATP and GroES in a fully alternating mechanism, although in this case, the folding protein would not have the benefit of encapsulation.

## The physiological relevance

It is evident, as discussed above, that at least *in vitro*, both types of complexes, symmetrical and asymmetrical, co-exist. Thus, the debate has changed its focus to the physiological relevance of the various forms observed. It has been well established that the velocity of the GroEL–GroES reaction cycle and the partitioning between various complexes depends on many important factors such as concentration and ratio of nucleotide, as well as concentrations of magnesium and potassium (reviewed in Grallert and Buchner, 2001; Sameshima et al., 2008, substrate protein Sameshima et al., 2010a; Yang et al., 2013; Ye and Lorimer, 2013; Fei et al., 2014, GroEL and GroES Azem et al., 1994b). The latter two are often expressed as ratios, but this could be misleading. In an *E. coli* cell under normal conditions, the concentration of GroEL is estimated to be ~35 μM protomer (Lorimer, 1996). This concentration can be even much higher under conditions of heat stress. To the best of our knowledge, most *in vitro* assays of GroEL are carried out at concentrations much <10 μM for the chaperonin. In most biophysical studies, the concentrations used are on the order of 1 μM and even much less. At these concentrations, we know that the chloroplast and mitochondrial chaperonins dissociate to monomers in the presence of ATP (Bloom et al., 1983; Lissin, 1995; Viitanen et al., 1998; Bonshtien et al., 2009). Since some oligomers or complexes may dissociate upon dilution, we cannot assume that we are working under the exact physiological conditions or that the species that we observe necessarily reflect those relevant to the cell. Moreover, the local concentrations of the above and other small effectors are difficult to determine *in vivo* in a precise manner, making it even more complicated to define the active species. Another factor that may affect the oligomeric state includes temperature (Goloubinoff et al., 1997; Llorca et al., 1998). One way of investigating the physiological relevance of folding intermediates would be to follow the reaction cycle *in vivo*, not an easy task at all. The only laboratory with a monopoly on physiological conditions is the cell itself. Until then, the significance of the *in vitro* experiments for the actual situation *in vivo* will remain an open question.

## Divergent mechanisms? insight from structural studies of the mitochondrial chaperonin

The human mitochondria harbor a type I chaperonin system (Hsp60), which is related, at least at the primary sequence level, to the bacterial machinery. Surprisingly, the mitochondrial chaperonin was isolated as single ring and it was traditionally regarded as active in this form (Nielsen and Cowan, 1998). However, subsequent studies using analytical ultracentrifugation and electron microscopy showed that the protein exists in dynamic equilibrium between single and double rings (Levy-Rimler et al., 2001; Vilasi et al., 2014). While Hsp60 is detected predominantly as a single ring, upon addition of ATP and mitochondrial co-chaperonin, the equilibrium is shifted toward formation of double-ringed, football shaped structures (Levy-Rimler et al., 2001). This observation again reinforces the relevance of working as close as possible to physiological conditions. However, at concentrations that are used routinely in the field, the mitochondrial chaperonin dissociates not only to single rings but also to 60 kDa monomers (Viitanen et al., 1998). The fact that most of the apo-protein is single ringed, even in the presence of bound substrate, while in the presence of ATP and co-chaperonin the protein oligomerizes to primarily the football form, presents an additional challenge to the prevailing theory of chaperonin function, since the complex does not even seem to pass through the asymmetric, bullet-shaped complex. Instead, a small amount of “half footballs” are observed- one ring of Hsp60 bound to one ring of Hsp10 (Viitanen et al., 1998). Similar structures were observed for the *Thermus thermophilus* chaperonin as well (Ishii et al., 1995). This suggests that the mitochondrial homolog may function using its own unique reaction mechanism, in which the tetradecamer exists as a football in its protein-folding state, but dissociates into two single rings at some point during the cycle (Figure 2C). Dissociation to single rings was observed previously both for mHsp60 (Levy-Rimler et al., 2002) and for Cpn60 from *T. thermophilus* (Todd et al., 1995; Taguchi, 2015). For mHsp60, hydrolysis of ATP to ADP was proposed to cause a drastic decrease in co-chaperonin binding, allowing rapid dissociation of the mitochondrial Hsp10 and release of the encapsulated protein (Nielsen and Cowan, 1998).

Additional evidence for a unique mechanism can be gleaned from the recent crystal structure of a mitochondrial Hsp60 variant in complex with Hsp10, which crystallized as a football complex that displays one subunit in a different conformation than the other six in the ring (Nisemblat et al., 2015). This is in stark contrast to GroEL, for which one hallmark of its mechanism is the high level of cooperativity between subunits in each ring, which results in their concerted movement (Saibil et al., 2013). Moreover, the crystallized mHsp60–mHsp10 structure shows ADP in all the 14 sites, a conformation which cannot exist for GroEL–GroES due to the strong inter-ring negative cooperativity of nucleotide binding. Finally, in this football structure, the surface contact area between the two rings is much more extensive than for the GroEL football or bullet (Nisemblat et al., 2015). Such an extensive interface is not consistent with the weak inter-ring interaction observed upon binding of ATP to the second ring of GroEL (Clare et al., 2012). Thus, a large body of evidence suggests that the mitochondrial chaperonin may have evolved a unique mechanism related to its specific functions. This mechanism seems to involve primarily football structures during the folding cycle that alternate with half footballs and single rings.

More recently, a novel phage-encoded Cpn60 was described which was also proposed to function via single ringed intermediates. In this case, the apo form of the chaperonin is tetradecameric. However, upon nucleotide binding, the oligomer dissociates into two heptameric rings with a largely expanded cavity, able to accommodate larger substrate proteins than other known chaperonins (Molugu et al., 2016). Thus, similar to what was proposed for the mitochondrial and *T. thermophilus* chaperonins, phi-EL seems to incorporate a single-ringed intermediate in its reaction cycle.

## Concluding remarks

Although a large body of data has accumulated concerning the chaperonin system and its mechanism of action, there are still a number of open questions concerning its reaction cycle(s) and the nature of the active species. The existence of different species in the functional cycle is now almost universally accepted and has paved the way for research into the role of each species in the molecular mechanism. Cutting-edge technologies applied to this system are allowing dissection of the protein folding events at the molecular level, describing how both symmetric and asymmetric species cooperate to facilitate protein folding. Investigation of GroEL homologs from different systems has also contributed interesting twists to the discussion of chaperonin mechanism. However, despite the wealth of research on the chaperonin system, most studies to date have been carried out *in vitro* on the *E. coli* GroEL and GroES. It will be intriguing to examine in depth the mechanistic divergence of organellar chaperonins from the *E. coli* paradigm at the molecular level and try to understand what advantages they provide to their respective systems. Analysis of the mitochondrial Hsp60 has already highlighted involvement of the symmetric football structure in the reaction cycle, as well as possible half-footballs. It will also be interesting to analyze intermediates in the highly complex chloroplast chaperonin system, for which multiple homologous products are expressed for both the GroEL- and GroES-like genes, forming a variety of labile hetero-oligomeric complexes *in vitro*.

## Author contributions

CW, AA, SN, and FJ wrote the paper. FJ and SN designed the figures.

## Funding

This work was funded by the Israel Science Foundation (ISF-1507/13) and the United States - Israel Binational Science Foundation (BSF-2015214).

### Conflict of interest statement

The authors declare that the research was conducted in the absence of any commercial or financial relationships that could be construed as a potential conflict of interest.
